# Optimal 2D-SIM reconstruction by two filtering steps with Richardson-Lucy deconvolution

**DOI:** 10.1038/srep37149

**Published:** 2016-11-16

**Authors:** Victor Perez, Bo-Jui Chang, Ernst Hans Karl Stelzer

**Affiliations:** 1 Buchmann Institute for Molecular Life Sciences (BMLS) Goethe Universität Frankfurt am Main Max-von-Laue-Strasse 15, 60438 Frankfurt am Main, Germany

## Abstract

Structured illumination microscopy relies on reconstruction algorithms to yield super-resolution images. Artifacts can arise in the reconstruction and affect the image quality. Current reconstruction methods involve a parametrized apodization function and a Wiener filter. Empirically tuning the parameters in these functions can minimize artifacts, but such an approach is subjective and produces volatile results. We present a robust and objective method that yields optimal results by two straightforward filtering steps with Richardson-Lucy-based deconvolutions. We provide a resource to identify artifacts in 2D-SIM images by analyzing two main reasons for artifacts, out-of-focus background and a fluctuating reconstruction spectrum. We show how the filtering steps improve images of test specimens, microtubules, yeast and mammalian cells.

Since the introduction of structured illumination microscopy (SIM) as a super-resolution imaging technique a reconstruction algorithm has been required^1^,^20^. The tasks of the reconstruction algorithm are threefold. First, the extraction of the domains 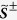 that contain the high resolution information. Second, the extraction of the reconstruction parameters, namely the spatial frequency 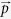 and the phase 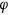 of the illumination pattern. Finally, the correct placement of the domains 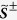 in Fourier space to assemble the spectrum 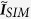 of the super-resolution image 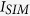. The first task is carried out by solving a linear system of equations. The reconstruction parameters are usually estimated, either through cross-correlations or through a linear regression^1^,^2^, using the information redundancy between the high frequency domains 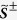 and the diffraction limited frequencies 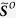. To achieve a correct assembly of 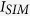, an accurate estimate of 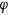 and 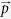 are important as they determine the solutions of 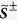 and their correct placement respectively. Consequently, most publications introducing reconstruction methods are set in a context where the values of 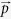 and 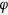 are optimized in order to avoid artifacts due to a wrong estimate^2^,^21^,^22^,^23^,^24^.

We analyze the out-of-focus background and fluctuations in the image spectrum as important reasons for artifacts. The out-of-focus background generates periodic patterns in the image background^3^,^4^, while ripples in the image spectrum spuriously highlights or attenuates certain frequencies that translate into side lobe artifacts that diminish the quality of the super-resolved image 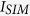. Our analysis leads to a reconstruction approach where we implement a pre- and a post-reconstruction filtering steps to minimize the artifacts. The initial filtering is performed on the raw images. It prevents the artifacts originating from the out-of-focus signal and facilitates the recovery of 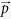, even when it lies beyond the cut-off frequency of the detection objective. The second filtering is performed on the reconstruction 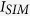 and compensates the uneven weighting of 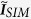, yielding a smoother spectrum that improves the high frequency details in the reconstruction.

In the most commonly applied SIM reconstruction procedure proposed by Gustaffson *et al*.^1^, the domains 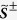 are combined through a Wiener filter while an apodization function is used to remove the ringing artifacts produced by this filter. The apodization function has evolved into a parametrized function whose shape is empirically tuned to enhance the reconstruction and eliminate artifacts. In this sense, the reconstruction has become reliant on the appointed apodization function and is still a heavily debated issue^2^,^3^,^4^,^5^,^6^,^7^,^8^,^9^. In our method, we perform two filtering steps with the Richardson-Lucy (RL) deconvolution. The advantage of this deconvolution algorithm over the Wiener filter is that it intrinsically accounts for the Poisson noise characteristic of the photon counting process in cameras.

In our reconstruction approach we completely prescind from any parameter tuning and only rely on the unbiased filtering steps to reduce artifacts and produce user-independent 2D-SIM reconstructions. We demonstrate the positive effects with images of diverse biological samples acquired with our set-up and an external data set.

## Results

### Two-dimensional SIM super-resolution reconstruction rationale

In 2D-SIM a sample is illuminated with a sinusoidal pattern along different orientations. The acquisition process uses three different orientations and at least three phases for each orientation, i.e. a minimal set of nine images per plane. If the period of the pattern is *T*_*o*_, its spatial frequency is given by 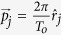. The pattern orientation is determined by the unit vector 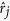 (*j* = *1, 2, 3*). The three pattern phases for each *j* orientation are denoted as 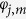. Although these phases can be arbitrary, uniform steps are preferred to achieve uniform illumination of the whole sample. Thus the phases are equally distributed within the [0, 2π] interval, 
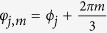
; (*m* = *0, 1, 2*), with 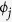 representing the initial phase of the pattern in the *j* orientation. The intensity profile of a pattern at a given point 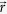 in space is:


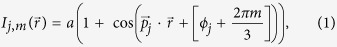


with a constant *a* proportional to the illumination intensity.

**Figure 1:**
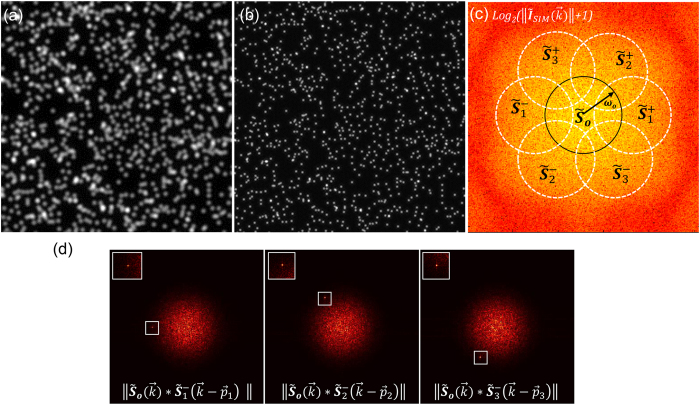
Simulation of 2D structured illumination with fluorescent beads. (**a**) Wide-field image. (**b**) I_SIM_ reconstruction. (**c**) Logarithm of the *I*_*SIM*_ power spectrum: 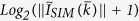. (**d**) Cross-correlations of extended domains with the central one, 
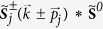
, produce a sharp peak due to their information overlap. The peak provides an estimate of the reconstruction parameter 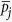. The regions where the peak appears are enclosed in the white box, top left corner insets show zooms of those regions.

The images in the 9-tuple recorded in each plane are represented by 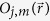 and Fourier transforms by 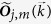. The linear system in equation (2) relates 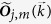 with domains 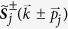 of the object/sample spectrum that are not accessible in conventional wide-field microscopy. These domains contribute new spatial frequencies, which in turn increase the resolution by extending the effective support of the microscope’s optical transfer function (OTF) (see Fig. 1). Solving equation (2) provides the extended domains 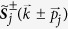 as well as the central domain 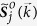 that contains the diffraction limited information^20^.


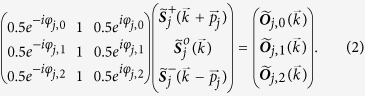


The set of extended domains 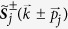 is available due to the intensity modulation provided by the illumination pattern. As explicitly stated in their argument they are shifted into the support of the OTF by the vectors 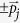. Therefore, when a super-resolution image is assembled, the extended domains have to be shifted to their correct location in Fourier space by the vectors 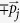. The super-resolution image 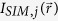 is then given by equation (3), where the shifting properties of the Fourier transform have been employed to shift the extended domains through the exponential factors.


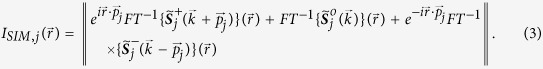



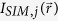
 will yield an image with a major resolution gain in the *j* direction. To gain resolution along the other orientations, the extended domains along each *j* orientation have to be calculated from equation (2) and then assembled using the following equation:


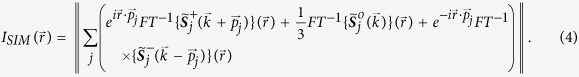


A typical footprint of an 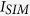 image spectrum 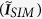 is shown in Fig. 1c. The approximated perimeter of the extended domains is outlined in white and the central domain in black. The cut-off frequency 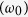 of the OTF determines the radius of all domains. The resolution gain in 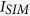 is determined by the content of spatial frequencies in 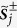 beyond the cut-off frequency.

The spectrum seen in Fig. 1c corresponds to a simulated 2D-SIM image acquisition of 1000 fluorescent beads 115 nm in diameter. The illumination pattern was simulated with equation (1) using a period of *T*_*o*_~260 nm, *a* = 1000 and patterns oriented along 0°, 60° and 120°. All orientations have the same initial phase 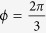. The pixel size is 57.6 nm, the emission wavelength is 515 nm and the detection system is a 63x/NA 1.0 water immersion objective lens. The pixel size, emission wavelength and detection parameters were chosen to resemble the experimental conditions in our coherent structured illumination light sheet-based fluorescence microscope (csiLSFM), which creates an illumination pattern by interfering two light sheets (Supplementary Material Fig. S1)^14^.

### Correlation as a tool for reconstruction optimization

Good estimates of the pattern spatial frequency 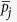
and the pattern’s initial phase 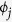 are essential before reconstructing an image with equation (4). The spatial frequency 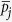 accomplishes the correct translation of the extended domains. The initial phase 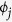 defines the coefficients 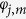 of the linear system in equation (2) and determines the correct solutions for the extended domains.

There is usually a certain extent of overlap between the central 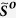 and the different extended 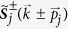 domains. It is this overlap of information that can be used to estimate the pattern spatial frequency 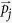. The cross-correlation of the central domain with an extended domain,


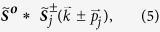


leads to a peak where the information overlap is maximal. The peak position provides a good estimate of 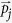. Figure 1d illustrates this situation by correlating some of the domains in Fig. 1c. The cross-correlation in equation (5) has not only been used to estimate 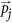 but also to optimize the values of the phases 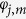
2. We extract the initial phases 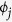 by carrying out a normalized cross-correlation between the reconstruction 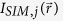
and the wide-field image. This approach evaluates the similarity of the features contained in both images.

### Pattern spatial frequency estimation

Before carrying out the correlation between 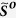 and 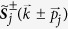, one must obtain these domains by solving equation (2). Their solutions for 
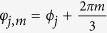
, (*m* = *0, 1, 2*) are:


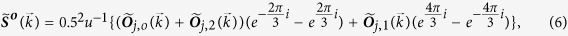



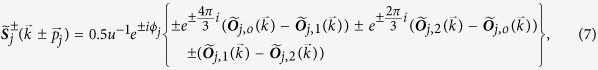


where 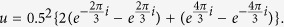


Equations (6 and 7) offer information for the reconstruction: First, 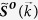 is not a function of the initial phase *ϕ*_*j*_, and second, the solution of 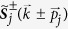 incorporates *ϕ*_*j*_ in an exponential factor. Therefore, *ϕ*_*j*_ only acts as a scaling factor to the norm of the correlation 
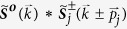
. The position of the peak that determines 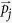 is independent of the chosen initial phase in equation (2). Consequently, we arbitrarily pick a value of the initial phase *ϕ*_*j*_ and then solve equation (2) to obtain 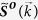 and 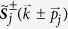.

The spatial frequency 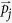 is calculated as:


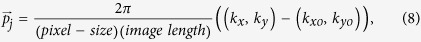


The pixel-size is given in units of length, image length is the number of pixels along one axis of a square grid, (*k*_*x*_,*k*_*y*_) is the peak coordinate and 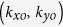 is the grid center coordinate.

### Pattern phase estimation through reconstruction evaluation

**Figure 2:**
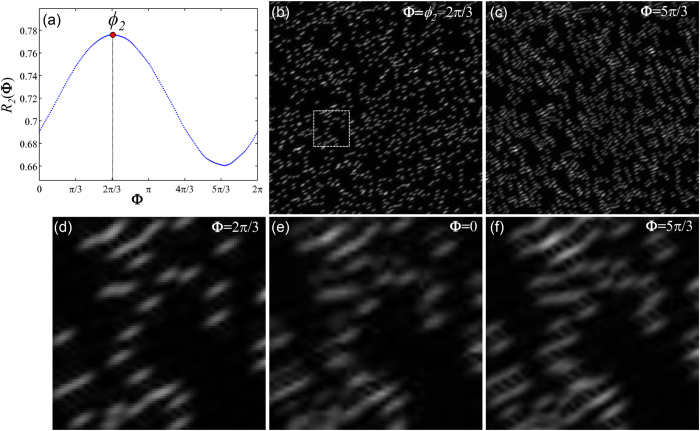
Estimation of initial phase *ϕ*_*j*_ by correlating the reconstruction *I*_*SIM,j*_ and the wide-field. (**a**) The *R*_*2*_(**Φ**) curve provides the values of the normalized cross-correlation between the wide-field and the reconstruction *I*_*SIM,2*_ at different phases from 0 to 2π. Arguments of the maximum and minimum of the curve respectively determine the best and worst estimates of the initial phase. *I*_*SIM,2*_ reconstruction with (**b**) 2π/3 and (**c**) 5π/3. Taking the former as reference it is clear that the latter is full of artifacts. Magnifications of the area within the white rectangle: Reconstructions using different initial phase values (**d**) 2π/3, (**e**) 0 and (**f**) 5π/3. The degradation of the reconstruction seems gradual, from the best result in (**d**), then to a stage of moderate artifact occurrence in (**e**) and finally severe distortions in (**f**).

So far, we established that the value of the initial phase is not important to recover the spatial frequency 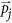 from equation (5), yet its correct value is essential for a good super-resolution reconstruction. The reason is that the exponential factor in equation (7) determines the phase of the image through its imaginary argument, it is well known that the image phase is key in the perception and preservation of image features in the real space^10^. In Fig. 2a,b the sample introduced in Fig. 1a is reconstructed for the 60° orientation 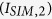 using the correct initial phase 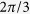 and an incorrect phase 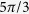. The degradation of the reconstruction using the wrong initial phase (Fig. 2b) is obvious since features that are not original appear in the image.

To recover the correct value of the initial phase 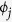, we perform a normalized cross-correlation between the reconstruction 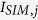 and the wide-field *I*_*o*_ (equation (9)). This correlation is only evaluated at the origin (*x*_*o*_, *y*_*o*_), i.e. at offset zero, and it is carried out for each of the values in a given discrete interval 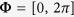. Hence, a reconstruction 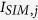 is performed for each of the values in 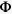, by considering them as possible initial phase values to solve equation (2). Then each reconstruction follows equation (9) and the cross-correlation value is allocated in *R*_*j*_, which becomes a function of 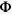. The correct initial phase 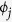 is then given by the argument that produces the maximum value of *R*_*j*_.


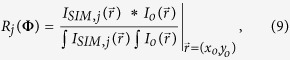


The cross-correlation compares 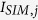 with the diffraction limited image *I*_*o*_, which is calculated using 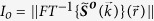. The rationale of using equation (9) to estimate the initial phase is that it also works as an assessment step for the reconstruction, since, even though 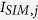 has a higher resolution, it should still share similar features with the ground truth established by *I*_*o*_. Therefore, a good 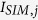 reconstruction is maximally correlated with *I*_*o*_. On the other hand, using a wrong estimate of 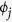 leads to a distorted reconstruction reducing its similarity with *I*_*o*_, which in turn causes a drop in the value of the correlation.

**Figure 3:**
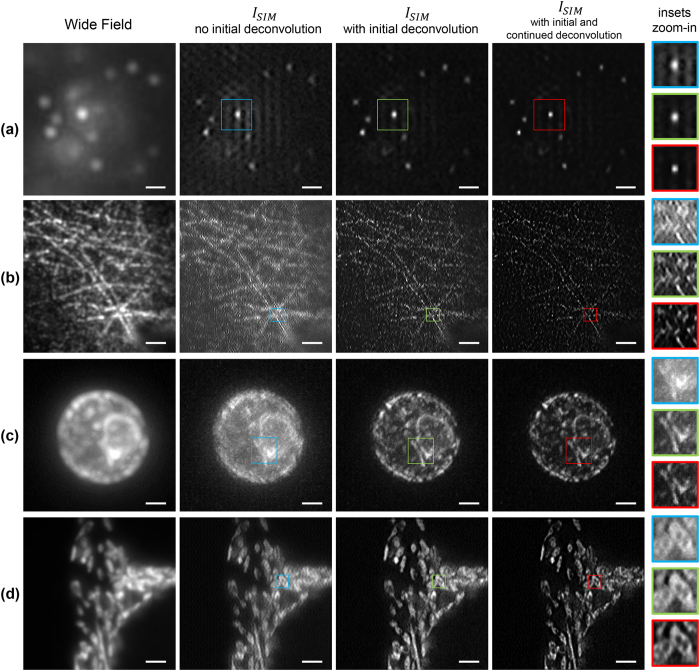
Comparison of SIM image reconstruction of different samples with/without the initial and continued deconvolution steps. All samples are excited with a 488 nm laser, while a bandpass filter (FF02-525/50-25, Semrock) is used in the detection path. (**a**) Fluorescent beads are 40 nm (F8759, Invitrogen) in diameter. (**b**) HUVE cells with α-tubulin immuno-stained with Alexa 488. (**c**) Maximum intensity projection of a 7 μm image stack of a living wild type yeast cell (BY4741, transformed with pRS415‐ERsfGFP‐HDEL)^11^. The endoplasmic reticulum is GFP-tagged and the sample is embedded in 1.5% low melt agarose. (**d**) Hepatocellular carcinoma cells (HepG2 cell line) on a coverslip expressing GFP in mitochondria. The period of the illumination patterns in (**a**,**d**) is around 300 nm and, 215 nm and 240 nm for (**b**,**c**) respectively. In each reconstruction, a region was selected and enclosed in a color box. Magnifications of these regions are displayed in the rightmost column. Scale bar: 2 μm except for (**a**) 600 nm.

A curve *R*_*2*_(**Φ**) is displayed in Fig. 2a corresponding to the 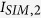 reconstruction of our sample in Fig. 1a. In this case, **Φ** is a regular interval consisting of 200 points. A red dot marks the position of the maximum in the curve, which, as expected, is located at 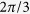. The minimum value occurs at 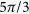 associated to the “worst” reconstruction (see Fig. 2c). Magnifications of the area enclosed in the white square (Fig. 2b) are shown for values of 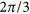, 0 and 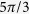 (Fig. 2d–f). Anisotropy of the beads is expected, since we consider just one orientation for the reconstruction. Please notice, how the reconstruction using the correct phase (Fig. 1d) becomes degraded when wrong initial phases are used (Fig. 1e,f). This fact is also reflected in the *R*_*2*_(**Φ**) curve with the values of the correlation dropping around 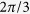. This approach to estimate 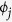 was tested on both sparse and dense samples giving good results as demonstrated with the successful reconstructions of the α-tubulin and mitochondria samples (Fig. 3b,d third and fourth columns). Each 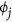 was estimated independently by its corresponding 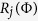 curve (see Supplementary Figure S6).

### Initial deconvolution of *O*_*j,m*_ reduces artifacts and enhances detection of reconstruction parameters

A real wide-field image always contains noise and out-of-focus fluorescence. Therefore, the cross-correlations in equations (5 and 9) are affected, which in turn makes the retrieval of the pattern spatial frequency and its initial phase difficult.

**Figure 4:**
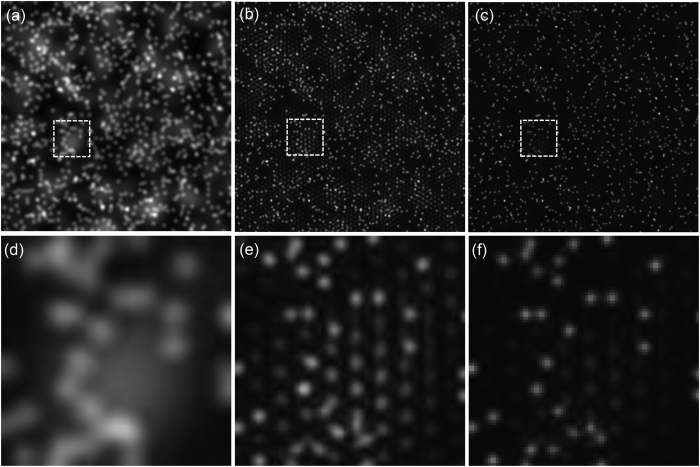
Using raw images *O*_*j,m*_ with out-of-focus background yields *I*_*SIM*_ reconstructions with artifacts in the background regions. If the raw *O*_*j,m*_ images are deconvolved prior to the reconstruction, no artifacts are visible in the reconstructed image. (**a**) Wide-field image of simulated beads with out-of-focus fluorescence background. (**b**) *I*_*SIM*_ without the initial deconvolution, notice the occurrence of periodic artifacts in the regions where the wide-field image presents prominent out-of-focus fluorescence. (**c**) *I*_*SIM*_ applying the initial deconvolution step, artifacts do not come up in this reconstruction. (**d**–**f**) show respectively magnifications of the white outlined areas in (**a**,**b**,**c**).

This issue has been addressed in ref. 2 where weighted cross-correlations have been used to diminish the effects of noise. The applied weighting functions, which rely on signal-to-noise ratios, were obtained either empirically or experimentally. On the other hand, weighting functions do not address the out-of-focus fluorescence issue directly, which is important in thick samples, and if not removed becomes a major source of artifacts. We experimentally observed the artifacts as quasi-periodic patterns (Fig. 3/second column) and also verified this effect with simulations (Fig. 4b). As Fig. 4 shows, these patterns appear in the regions where strong background fluorescence is present. The blurry regions in the wide-field image (Fig. 4a,d) directly relate to the occurrence of artifacts in the same regions of the 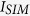 reconstruction (Fig. 4b,e). Similar artifacts have been previously reported and associated to raw images (*O*_*j,m*_) with low signal-to-noise ratio and strong out-of-focus signal^3^,^4^,^12^.

We propose a straightforward approach to remove artifacts in the reconstruction related to the out-of-focus fluorescence. We implement the Richardson-Lucy algorithm to deconvolve the set of *O*_*j,m*_ images with the PSF of the imaging system prior to solving equation (2). Additionally, this initial deconvolution diminishes the weighting effect of the OTF and enhances the detection of the peak resulting from the cross-correlation in equation (5). This enhancement is important especially when the frequency of the structured illumination pattern is beyond the diffraction limit since the overlap between 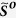 and 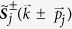 becomes small.

### Initial deconvolution of *O*_*j,m*_ reduces out-of-focus signal related artifacts in 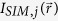


The reduction of artifacts due to the initial deconvolution is illustrated in Fig. 4. Out-of-focus fluorescence has been simulated in our synthetic sample shown in Fig. 1a by adding a layer of 200 randomly distributed out-of-focus beads 115 nm in diameter. These beads are blurred by a PSF four times larger than the PSF of the emission wavelength (515 nm) of the in-focus beads. Structured illumination was simulated with the illumination and detection conditions summarized above. A set of *O*_*j,m*_ images is obtained and used to reconstruct 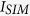. The wide-field and the 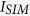 images are shown in Fig. 4a,b, respectively. A second reconstruction was carried out but deconvolving all *O*_*j,m*_ images with 10 iterations of the Richardson-Lucy algorithm and the PSF of the detection system. Comparing the reconstructions in Fig. 4b,c proves that the initial deconvolution step suppresses the artifacts due to the out-of-focus fluorescence and provides a cleaner background. The effects on 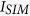 with and without the initial deconvolution are not just noticeable in simulations but also in experiments as compared in the second and third columns of Fig. 3. Non-initially deconvolved raw images produce reconstructions with seemingly periodic artifacts imprinted in the regions with background fluorescence, while applying the initial deconvolution step produces reconstructions with no artifacts.

When processing 3D stacks reconstructed in a plane-by-plane manner a 2D deconvolution is still preferred over a 3D deconvolution, since the latter appears to result in deconvolution related artifacts due to the non-applicability of spatial shift invariance (Supplementary Figure S8).

### Initial deconvolution enhances the detection of 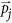


**Figure 5:**
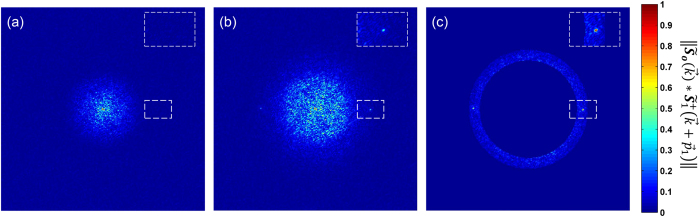
Initial deconvolution improves the estimate of the 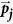 parameter by enhancing the peak occurrence in the correlation 
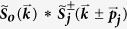
. Correlation result for a sample (**a**) without and (**b**) with initially deconvolved raw data *O*_*j,m*_. In the first case, there is no peak in the region where 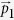 is expected (white dashed inset). In the second case the occurrence of the peak is clearly visible. (**c**) If an annular mask is applied to the correlation in (**b**) to highlight the region of interest, the peak can be extracted automatically by detecting the maximum in this region.

The initial deconvolution not only reduces periodic artifacts but it also facilitates the detection of the peak that determines 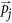. Especially for those data sets that have been illuminated under patterns with periods beyond the diffraction limit of the detection objective. This situation is illustrated in Fig. 5 where the norm of the cross-correlation of 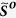 and 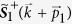 is displayed without (Fig. 5a) and with initial deconvolution (Fig. 5b). Both images were normalized to have a maximum value of one for comparison. The results shown in Fig. 5 were calculated from the dataset of a human umbilical vein endothelial cell (HUVEC) stained with α-tubulin (Fig. 3b and Supplementary Material Fig. S6). The dataset was recorded with our csiLSFM set-up, with an illumination pattern of 215 nm in period, meaning that the norm of its spatial frequency 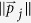 was 1.2 times larger than the cut-off frequency of our detection lens.

The white dashed insets in Fig. 5 indicate the region where the peak is expected but is not detectable in Fig. 5a. The situation is different for the initially deconvolved data since a local maximum is clear in Fig. 5b. An automated detection of the peak is implemented by defining a region of interest (ROI), which makes the peak the global maximum in the cross-correlation of 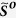 and 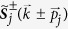. This ROI is a ring-shaped mask of e.g. 0.9*ρ* internal radius and 1.1*ρ* external radius, in which the mask has a value of 1 and 0 everywhere else. *ρ* denotes an expected estimate of the illumination pattern spatial frequency 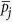. Figure 5c shows the mask applied to the image of Fig. 5b. *ρ* is estimated from a calibration curve, e.g. in our set-up the spatial frequency is a function of the mirror angular displacement α (Supplementary Material Fig. S2). The calibration curve needs to be determined only once, upon system alignment and by using small fluorescent beads as test specimens.

The main mechanism, by which initial deconvolution enhances the peak occurrence is that deconvolution of the raw images increases their spectrum extent, which in turn increases the overlap of the 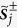 and 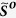
domains. A more fundamental explanation is that the deconvolution increases the effective modulation of the raw images by enhancing the differences between the raw images. Such enhancement facilitates the extraction of the spatial frequencies that form the extended domains (Eq. 7, Supplementary Figure S11). This becomes a feature when 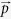 is larger than the cut-off frequency of the detection objective, since the overlap area of the domains decreases as 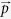 increases.

### Continued reconstruction deconvolution further enhances image quality

**Figure 6:**
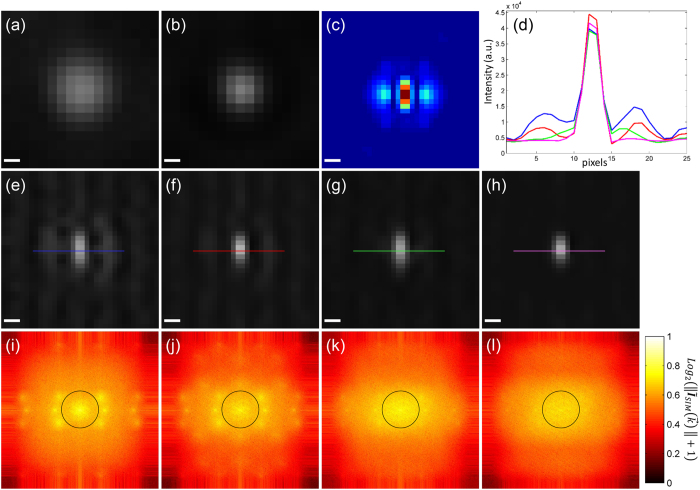
Continued deconvolution improves the reconstructed image *I*_*SIM*_. A single bead taken from a larger image consisting of 40 nm fluorescent beads is selected to show the effects of the initial and continued deconvolution on the reconstruction. (**a**) Wide-field, (**b**) deconvolved wide-field and (**c**) *PSF*_*SIM*_. Reconstructions (**e**) without initial nor continued deconvolution, (**f**) without initial but with continued deconvolution, (**g**) with initial but without continued deconvolution, (**h**) with initial and continued deconvolution. Their corresponding spectra are shown respectively in (**i**–**l**). A black circle in the spectra outlines the rim of the OTF. Seven spots stand out in (**i**,**j**) corresponding to the centers of the extended and central domains. This prominence generates unequal spectra and side lobes in the reconstructed bead. The continued deconvolution equalizes the spectrum generating correct reconstructions. The effects of the initial and continued deconvolutions are summarized in the intensity profiles in (**d**). The reconstruction with initial and continued deconvolution eliminates the side lobes effectively (magenta solid line). Scale bar: 100 nm. Pixel size: 28.8 nm.

Although we increased the image resolution by adding the extended domains to the central domain in equation (4), this operation might not yield optimal results as it induces different frequency weights in different regions of the spectrum due to the arithmetic addition. For example, the overlap of 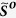 and 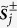 is large with smaller resolution gains, so the assembled spectrum has a major weighting in the overlap region. The low frequency content of the reconstruction is then emphasized. On the other hand, a high resolution gain implies less overlap of 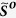 and 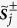. Such reduced overlap generates a fluctuating profile with valleys where the information drops due to the lower weighting at the edges of the OTF (Fig. 6i and Supplementary Material Fig. S4). This information drop in the spectrum translates into side lobes in the real space image (Fig. 6e), an effect which is especially obvious in high resolution gains (>2)^5^,^13^,^17^. Therefore, further improvement of the SIM reconstruction 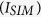 can be achieved by adjusting the weight of certain frequencies. This step is usually performed by multiplying the spectrum 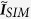 by an apodization function to tune the final appearance of the reconstruction^3^,^5^,^6^ (Supplementary Material Figures S3 and S5). In our reconstruction approach we simply deconvolve 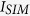 with an effective point spread function (*PSF*_*SIM*_), to equalize the weights of the high frequency and low frequency contents in the final image. The *PSF*_*SIM*_ is formed by applying equation 4 to the *PSF* of the microscope and the obtained set of 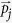:


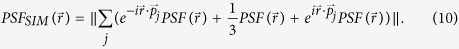


The deconvolution uses at most five iterations the Richardson-Lucy algorithm. How the reconstruction is improved by the continued deconvolution is demonstrated in Fig. 6 with 40 nm fluorescent beads and an illumination pattern with a period of 183 nm (wide-field Fig. 6a). The spectrum of a reconstruction, without undergoing initial or continued deconvolution, presents seven prominent spots (Fig. 6i). The prominence of such regions results in side lobes around the bead in the reconstruction (Fig. 6e). The unequal weighting of such a spectrum is slightly equalized after applying the continued deconvolution (Fig. 6j) and results in the reduction of the side lobes (Fig. 6f). Better results are achieved when the reconstruction is carried out using initially deconvolved raw images (Fig. 6g). The spectrum of this reconstruction (Fig. 6k) has a smoother profile than the ones in Fig. 6i,j. Best reconstruction results are obtained when using both the initial and continued deconvolution (Fig. 6h), there the side lobes are practically removed as validated by the intensity profile plot (Fig. 6d magenta line). The spectrum also has a more balanced profile without any sudden increases or decreases of values (Fig. 6l).

The continued deconvolution maximizes the resolution gain by equalizing both the low and the high spatial frequency content (Supplementary Figure S10). Its application to the reconstructions of biological sample is shown in the fourth column of Fig. 3. The contrast of the images is highly improved allowing a better identification of features in the sample, in comparison to the ones without the continued deconvolution (third column in Fig. 3). For 3D stacks reconstructed plane-by-plane a 3D continued deconvolution can be applied to achieve a resolution gain along the axial direction (Supplementary Figure S9). The *PSF*_*SIM*_ for such 3D deconvolution is calculated with equation 10 using the 3D *PSF* of the detection objective.

### Algorithm implementation on csiLSFM images

**Figure 7:**
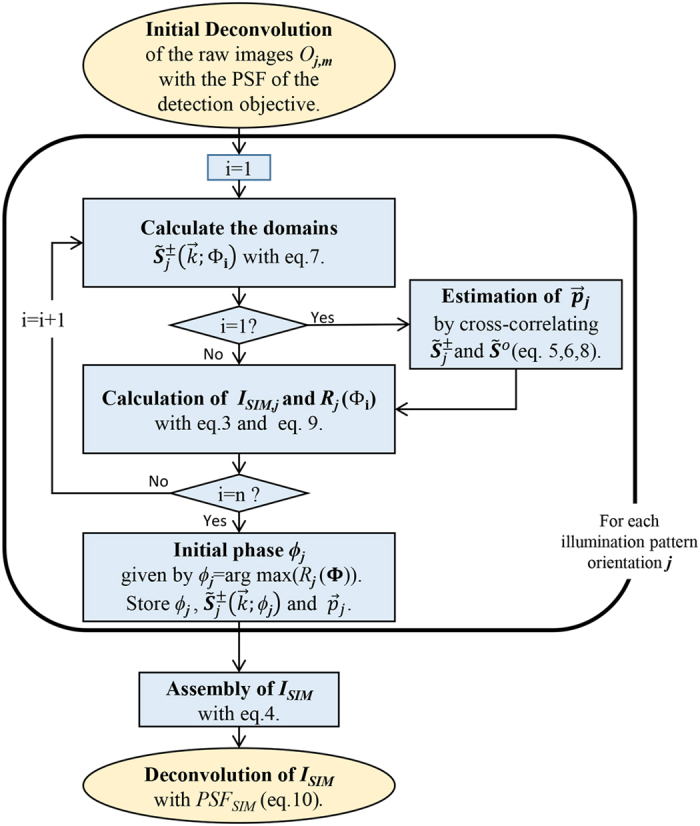
Flow chart of our reconstruction approach. For the Richardson-Lucy deconvolutions applied in the first and final steps we use no more than 10 and 5 iterations respectively. To estimate the initial phase *ϕ*_*j*_ an interval **Φ** = [0, 2π] containing *n* values must be defined. The values in the interval are denoted as Φ_*1*_, Φ_*2*_… Φ_*n*_. The correct reconstruction parameters and extended domains are extracted for each of the illumination pattern orientations and then used to assemble the super-resolved image *I*_*SIM*_.

We summarize our reconstruction method in the flow chart in Fig. 7. MATLAB code based on it has been scripted. The code has been used successfully in 2D samples and 3D samples reconstructed plane-by-plane (Fig. 3). For each plane an input of 9 raw images is required, reconstructing a plane of 256x256 pixels takes about 10 seconds when using an interval **Φ** of 200 points, in a CPU equipped with a 3.1 GHz processor and 16 GB RAM. The reconstruction time is reduced to three seconds if only 30 points, which suffice for a good reconstruction, are used.

Several examples of applying our methodology in SIM reconstruction are shown in Fig. 3. Images were taken with our csiLSFM set-up where the diffraction limit of the detection objective is approximately 260 nm. Independent of the nature of the sample, one can track the quasi-periodic artifacts due to the out-of-focus blur visible in the wide-field images (first column) in the reconstructions (second column). These artifacts do not arise when the initial deconvolution is applied, as demonstrated by the reconstructions in the third column. Finally, the reconstructions in the third column are further improved in contrast and resolution by applying the continued deconvolution. The improvement of the image due to the continued deconvolution step is particularly noticeable in Fig. 3d (red box). Many structures are now easily identifiable. Reconstructions of samples in Fig. 3b,c are noteworthy as the illumination patterns had periods of 215 nm and 250 nm respectively, i.e. frequencies 1.2 and 1.1 times larger than the cut-off frequency of the objective lens. These images confirm the application of our reconstruction methodology using pattern frequencies beyond the diffraction limit.

## Discussion

We introduce a robust and user-independent pipeline for reconstructing 2D-SIM images, i.e. it does not require the empirical tuning of any parametrized functions to achieve optimal reconstructions. Instead, we rely on a deconvolution applied to the raw images prior to the reconstruction process and on a further deconvolution performed on the reconstructed image. Both deconvolutions use the Richardson-Lucy algorithm, which is an automated iterative method that only requires an approximated PSF and is included in many software packages. Furthermore, the algorithm is very proficient in deconvolving SIM data sets as shown in^15^,^16^.

These two steps remove the commonly seen periodic and side lobe artifacts, which arise even when using a correct estimate of the reconstruction parameters. The first deconvolution also facilitates the recovery of illumination patterns with spatial frequencies beyond the diffraction limit. In our case, we were able to recover spatial frequencies of illumination patterns with 183 nm periods using an objective of 260 nm resolution (Fig. 6). In that case the resolution is improved by a factor of 2.4, which is more than the two-fold improvement available in conventional SIM, and consistent with TIRF-SIM microscopes where such fine patterns are also used^5^,^17^,^18^.

The main advantage of our method is that it directly leads to an optimal image by eliminating the artifacts with the deconvolution steps. As a result, our method is objective and reproducible. In contrast, a parameter tuning method does not remove such artifacts effectively and can lead to degraded images, depending on the set of chosen parameters (Supplementary Figure S5). Thus, requiring intense tuning to achieve a proper reconstruction. As an example of this situation, we show in Supplementary Figure S7 an image reconstructed with our method and three reconstructions produced by an ImageJ/Fiji plug-in based on the tuning of a Wiener filter and an apodization function^19^. After several attempts to optimize the image we were not able to achieve a reconstruction of the quality provided by our method. Additionally our method was tested in an external TIRF-SIM data set to demonstrate its compatibility with images produced by other SIM systems (Supplementary Figure S12).

We also applied the method to 3D-SIM data sets, leading to the same conclusions, i.e. objective reconstructions with reduced artifacts (Supplementary Figure S8). In summary, the proposed method provides an objective procedure that simplifies the image reconstruction task for researchers using SIM, especially the newly interested and non-experienced.

## Additional Information

**How to cite this article**: Perez, V. *et al*. Optimal 2D-SIM reconstruction by two filtering steps with Richardson-Lucy deconvolution. *Sci. Rep.* **6**, 37149; doi: 10.1038/srep37149 (2016).

**Publisher’s note**: Springer Nature remains neutral with regard to jurisdictional claims in published maps and institutional affiliations.

## Supplementary Material

Supplementary Information

## References

[b1] Gustafsson M. G. L. . Three-dimensional resolution doubling in wide-field fluorescence microscopy by structured illumination. Biophys. J. 94, 4957–4970 (2008). 18326650 10.1529/biophysj.107.120345 PMC2397368

[b2] Wicker K., Mandula O., Best G., Fiolka R. & Heintzmann R. Phase optimisation for structured illumination microscopy. Opt. Express 21, 2032–2049 (2013). 23389185 10.1364/OE.21.002032

[b3] Sahl S. J. . Comment on ‘Extended-resolution structured illumination imaging of endocytic and cytoskeletal dynamics’. Science (80-.). 352, 527 (2016). 10.1126/science.aad7983 27126030

[b4] Li D. & Betzig E. Response to Comment on ‘Extended-resolution structured illumination imaging of endocytic and cytoskeletal dynamics’. Science (80-.). 352, 527 (2016). 10.1126/science.aad8396 27126031

[b5] Beck M., Aschwanden M. & Stemmer a. Sub-100-nanometre resolution in total internal reflection fluorescence microscopy. Nanotechnology 232, 99–105 (2008). 10.1111/j.1365-2818.2008.02075.x 19017206

[b6] O’Holleran K. & Shaw M. Optimized approaches for optical sectioning and resolution enhancement in 2D structured illumination microscopy. Biomed. Opt. Express 5, 2580 (2014). 25136487 10.1364/BOE.5.002580 PMC4132990

[b7] Shaw M., Zajiczek L. & O’Holleran K. High speed structured illumination microscopy in optically thick samples. Methods 88, 11–19 (2015). 25839410 10.1016/j.ymeth.2015.03.020

[b8] Komis G. . Superresolution live imaging of plant cells using structured illumination microscopy. Nat. Protoc. 10, 1248–1263 (2015). 26203822 10.1038/nprot.2015.083

[b9] Watanabe K. . Structured line illumination Raman microscopy. Nat. Commun. 6, 10095 (2015). 26626144 10.1038/ncomms10095 PMC4686755

[b10] Oppenheim A. V. & Lim J. S. Importance of Phase in Signals. Proc. IEEE 69, 529–541 (1981).

[b11] Lajoie P., Moir R. D., Willis I. M. & Snapp E. L. Kar2p availability defines distinct forms of endoplasmic reticulum stress in living cells. Mol. Biol. Cell 23, 955–964 (2012). 22219379 10.1091/mbc.E11-12-0995 PMC3290652

[b12] Chu K. . Image reconstruction for structured-illumination microscopy with low signal level. Opt. Express 22, 8687 (2014). 24718238 10.1364/OE.22.008687

[b13] Chung E., Kim D. & So P. T. C. Extended resolution wide-field optical imaging: objective-launched standing-wave total internal reflection fluorescence microscopy. Opt. Lett. 31, 945–947 (2006). 16599220 10.1364/ol.31.000945

[b14] Stelzer E. H. K. Light-sheet fluorescence microscopy for quantitative biology. Nat. Methods 12, 23–26 (2014). 10.1038/nmeth.3219 25549266

[b15] Ingaramo M. . Richardson-Lucy deconvolution as a general tool for combining images with complementary strengths. ChemPhysChem 15, 794–800 (2014). 24436314 10.1002/cphc.201300831 PMC3986040

[b16] York A. G. . Instant super-resolution imaging in live cells and embryos via analog image processing. Nat. Methods 10, 1122–1126 (2013). 24097271 10.1038/nmeth.2687 PMC3898876

[b17] Fiolka R., Beck M. & Stemmer A. Structured illumination in total internal reflection fluorescence microscopy using a spatial light modulator. Opt. Lett. 33, 1629–1631 (2008). 18628820 10.1364/ol.33.001629

[b18] Kner P., Chhun B. B., Griffis E. R., Winoto L. & Gustafsson M. G. L. Super-resolution video microscopy of live cells by structured illumination. Nat. Methods 6, 339–342 (2009). 19404253 10.1038/nmeth.1324 PMC2895555

[b19] Müller M., Mönkemöller V., Hennig S., Hübner W. & Huser T. Open-source image reconstruction of super-resolution structured illumination microscopy data in ImageJ. Nat. Commun. 7, 10980 (2016). 26996201 10.1038/ncomms10980 PMC4802170

[b20] Gustafsson M. G. L., Agard D. A. & Sedat J. W. Doubling the lateral resolution of wide-field fluorescence microscopy using structured illumination. In BiOS 2000 The International Symposium on Biomedical Optics (eds Conchello J.-A., Cogswell C. J., Tescher A. G. & Wilson T.) 141–150 (International Society for Optics and Photonics 2000). doi: 10.1117/12.384189.

[b21] Mudry E. . Structured illumination microscopy using unknown speckle patterns. Nat. Photonics 6, 312–315 (2012).

[b22] Ayuk R. . Structured illumination fluorescence microscopy with distorted excitations using a filtered blind-SIM algorithm. Opt. Lett. 38, 4723–4726 (2013). 24322116 10.1364/OL.38.004723

[b23] Wicker K. Non-iterative determination of pattern phase in structured illumination microscopy using auto-correlations in Fourier space. Opt. Express 21, 24692 (2013). 24150313 10.1364/OE.21.024692

[b24] Shroff S. a, Fienup J. R. & Williams D. R. Phase-shift estimation in sinusoidally illuminated images for lateral superresolution. J. Opt. Soc. Am. A. Opt. Image Sci. Vis. 26, 413–424 (2009). 19183696 10.1364/josaa.26.000413

