# Supplementary material for: Optimal 2D-SIM reconstruction by two filtering steps with Richardson-Lucy deconvolution
Source: Sci Rep. 2016 Nov 16;6:37149. doi: 10.1038/srep37149 (PMC5111067; doi:10.1038/srep37149)
Supplement: Supplementary Information [file srep37149-s1.pdf]

Optimal 2D-SIM reconstruction by two filtering steps with Richardson-Lucy deconvolution

Supplementary information

Victor Perez, Bo-Jui Chang and Ernst Hans Karl Stelzer*

Buchmann Institute for Molecular Life Sciences (BMLS)

Goethe Universität Frankfurt am Main

Max-von-Laue-Strasse 15, 60438 Frankfurt am Main, Germany

Supplementary Figures

Fig. S1 csiLSFM set-up.

Fig. S2 csiLSFM calibration curve.

Fig. S3 Apodization function.

Fig. S4 High vs. low gain reconstructions with Wiener×Apodization and our reconstruction approach (RL reconstruction).

Fig. S5 Reconstruction dependence on the Apodization function parameters.

Fig. S6 Extraction of reconstruction parameters, $\vec{p}_j$ and ϕ_j, for the HUVEC data set.

Fig. S7 HepG2 data set reconstructed with the fairSIM implementation and our RL-reconstruction.

Fig. S8 Deconvolution artifacts when applying a 3D initial deconvolution to SIM stacks.

Fig. S9 3D continued deconvolution vs 2D continued deconvolution in plane-by-plane 2D-SIM reconstructions.

Fig. S10 Continued deconvolution enhances the reconstruction contrast.

Fig. S11 Initial Deconvolution enhances extraction of spatial frequencies.

Fig. S12 RL-reconstruction in external data set.

Table S1 Reconstruction parameters of Figure S7.

TableS2 Reconstruction parameters of Figure S12.

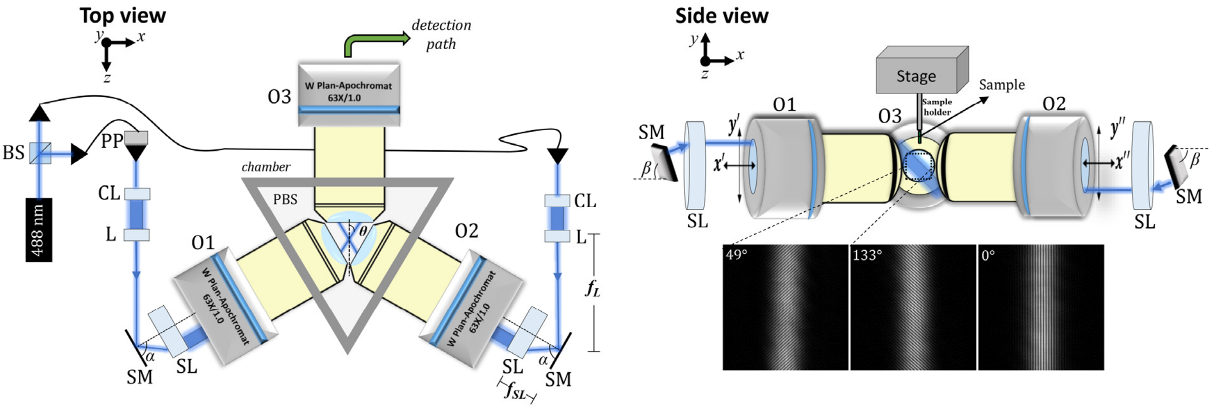

Figure S1 csiLSFM set-up. The beam splitter (BS) divides the laser beam into two coherent sources. Each beams is deflected individually into a polarization-preserving, single-mode fiber (kineFLEX, Qioptiq). The fiber collimators (F220FC-543, Thorlabs) at the end of the fibers deliver collimated beams and form two illumination paths. The chamber is filled with an appropriate medium, e.g. phosphate buffered saline (PBS), for biological specimens. The angle α defines the pattern period while β defines its orientation. PP: piezo nano-positioner, CL: cylindrical lens, L: achromatic lens, SM: scanning mirror, SL: f-θ lens, O1 and O2: illumination objectives, O3: detection objective.

Two objectives are used for illumination (O1,O2) and one for detection (O3), the three foci co-localize (Fig. S1). The illumination objectives are spread by 130° between their long axes. All objectives share the same specifications, i.e. water immersion, and a working distance of 2.1 mm (63x/NA1.0, W Plan-Apochromat, 421480-9900-000, Carl Zeiss). The output of each illumination objective is a light sheet shaped with a cylindrical lens (f=75 mm, F69-699, Edmund Optics) [1]. Interference of the two light sheets generates a two-dimensional sinusoidal pattern in the focal plane of the detection objective. The period T_o of the pattern is tuned by varying the half interference angle θ, which is a function of the angular displacement α of the scanning mirror (S-334.2SL, Physik Instrumente). In each illumination arm the achromatic lens (f=200 mm,

G322327322, Qioptiq) and the f-theta scanning lens (f=60.5 mm, S4LFT0061, Sill optics) form a 4f-telecentric system that projects the light sheet into the back focal plane of the illumination objective.

Rotation of the pattern is controlled by tilting the angle β in the scanning mirror, which correspondingly varies the height y' and y'' in each arm and anti-symmetrically tilts each light sheet on the focal plane of the detection objective. Three different orientations 0°, 49° and 133° of a pattern with T_o =301 nm are shown. The pattern phase is controlled with the piezo nano-positioner (P-725.4CD, Physik Instrumente) translating the optical fiber collimator.

Features of the csiLSFM are the flexible controls of the pattern period and orientation. It allows resolution gains larger than the two-fold in a usual SIM due to the decoupling of the detection and illumination paths. The two-fold constraint is inherent in epi-fluorescence configurations when using linear emissions since the period T_o of the pattern is restricted by the angular aperture of the objective. Maximum resolution gain is achieved when the two light sheets are counter-propagating (θ=90°). In our experimental conditions (λ_{exc}=488nm, n=1.333) that angle corresponds to an illumination pattern with a period of 183 nm.

The detection path (not shown) starts with O3 followed by an emission filter (FF02-525/50-25, Semrock) to block the laser for the observation of the fluorescence signal. A 1x tube lens (452960, Carl Zeiss) together with the objective is used to form the infinity corrected image. We use a CMOS camera with a pixel array of 1920x1440 and a pixel pitch of 3.63 µm (C11440, ORCA-Flash 2.8, Hamamatsu). The three objectives are partially inserted into a customized sealed polyoxymethylene chamber with an open top for the sample entry. The sample is embedded in phytagel, agarose, or a coverslip, and is mounted on a rod-like holder parallel to the y axis. The

holder is attached to a 4-axis (*xyz* translation and a rotation around *y*) motorized stage (custom-designed, SmarAct).

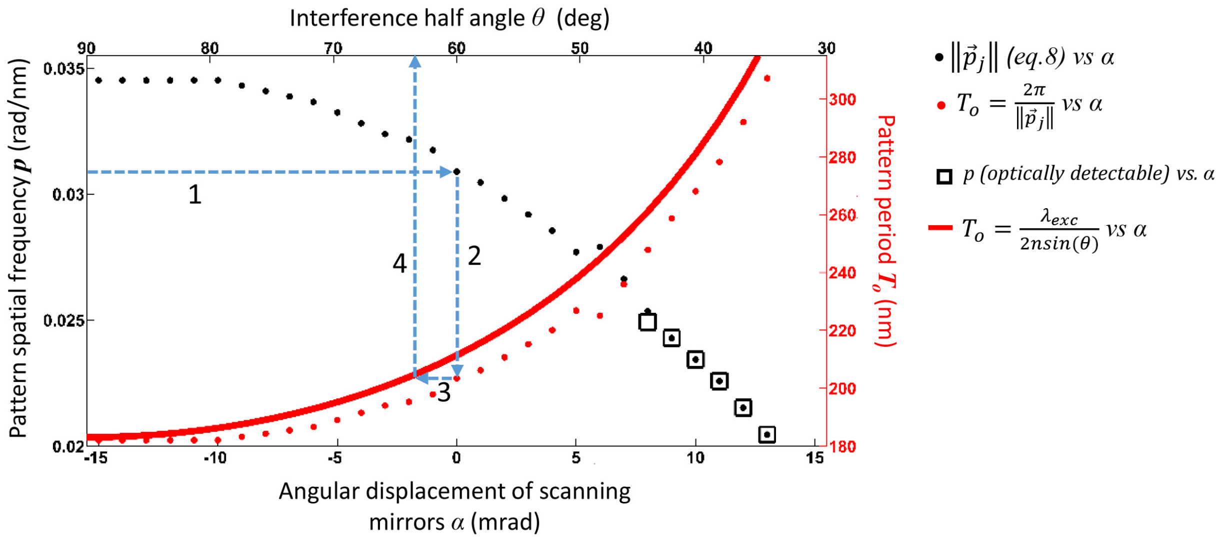

Figure S2 csiLSFM calibration curve. Images of beads are taken at different angular displacements α, and the norm of the spatial frequency $\vec{p}_j$ is calculated with equation 8 (black dots). Inverting the values of this curve times 2π yields the period T_0 (red dots). Comparing experimental data (red dots) with theoretical values (red solid line) allows an estimation of the half interference angle θ. The center of the black squares represents the spatial frequency values obtained from the range of angles α where the pattern is visible in the detection objective. A good match is found between these optically detected values and the cross-correlation estimates. Finally, from this calibration curve an estimated spatial frequency value expected for other samples can be obtained. The estimated spatial frequency corresponding to angular displacements α is used as the reference radius for the mask shown in Figure 5c.

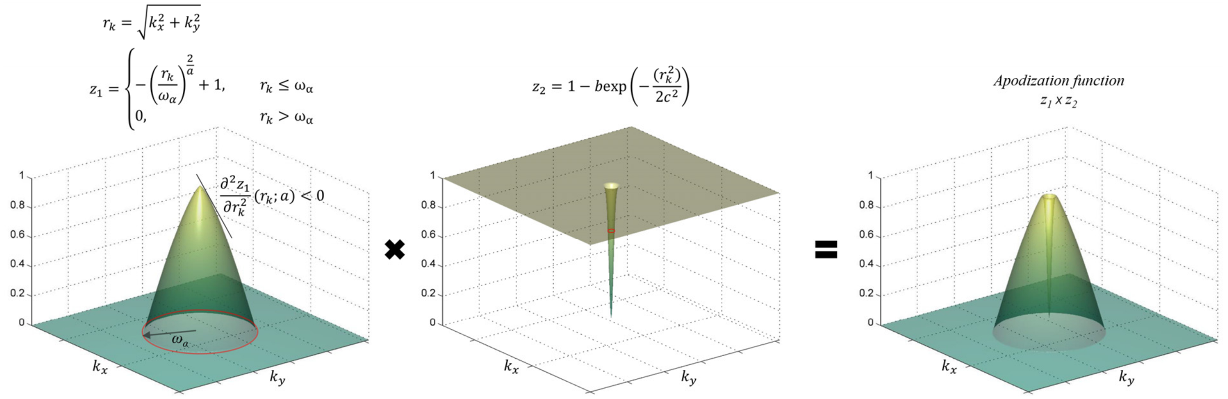

Figure S3 Apodization function applied to the spectra of the reconstructed image when using a Wiener filter to assemble the domains. This function is formed by the product of z_1 and z_2. z_1 cuts off the frequencies larger than the parameter ω_a, whose value is chosen to be around the expected largest frequency. Furthermore, the parameter a determines the concavity ($a<2$) or convexity ($a>2$) of the function surface. A concave surface enhances the high frequencies while a convex one hinders them. Proper tuning of the parameter a enhances the image quality by suppressing artifacts such as side lobes (Figure S4). z_2 is a function that suppresses out-of-focus signal by reducing the central value of the spectra. The parameter b defines the strength of the suppression and c determines the size of the suppressed area.

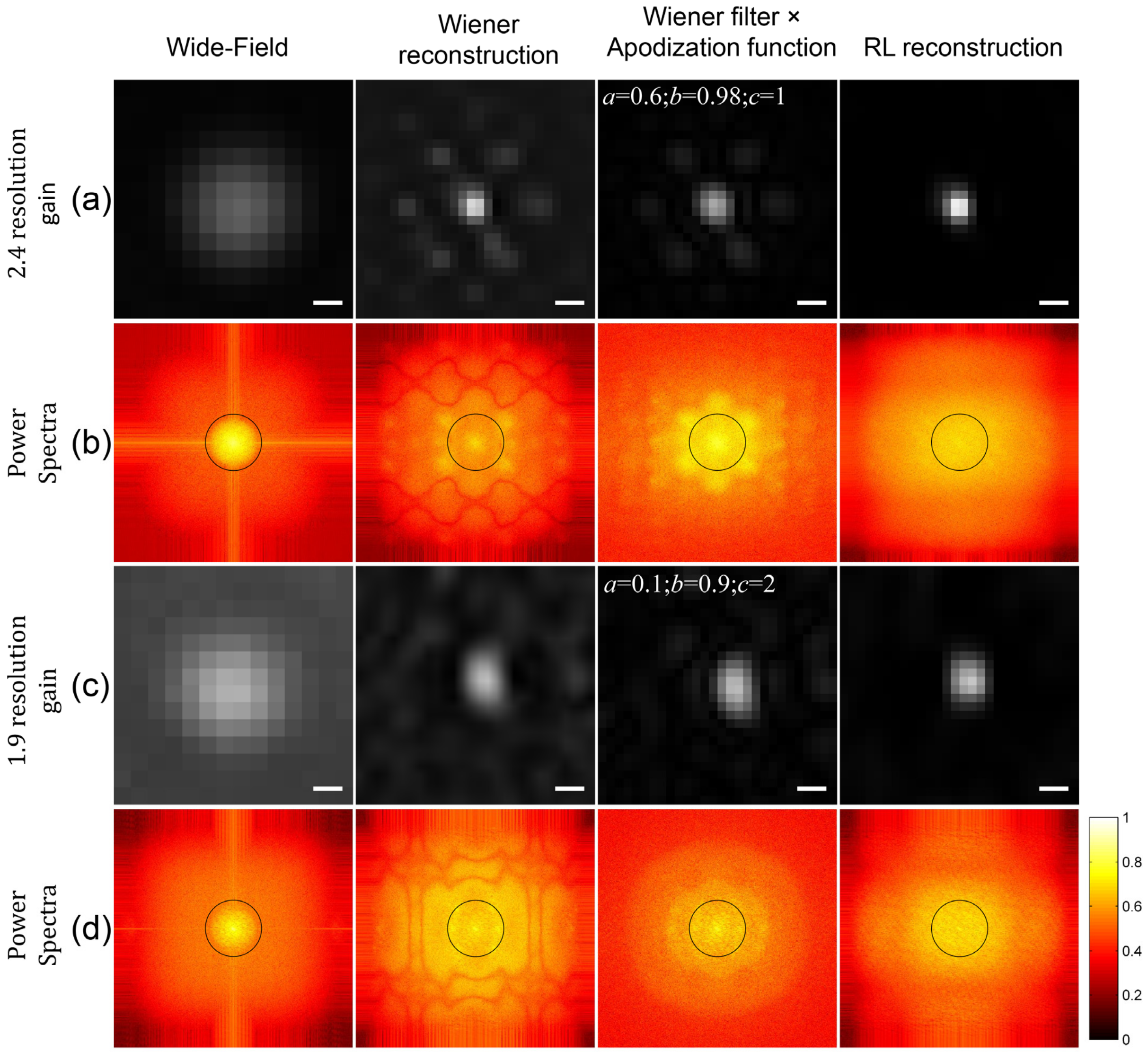

Figure S4 Reconstructions at high and low resolution gains. Comparison between reconstructions using the Wiener-filter×Apodization approach in references [2], [3] and our reconstruction method using the initial and continued deconvolution with the Richardson-Lucy algorithm (labeled as RL reconstruction). The comparison concerns the reconstruction effects on a point source, which is a bead cropped from a larger field of view with 40 nm fluorescent beads embedded in phytagel. **(a)** Wide-field and reconstructions of a sample illuminated with a pattern period of 183 nm to achieve a 2.4 resolution gain. Below each image is the corresponding **(b)** spectrum of the whole field of view. **(c)** Sample under a 307 nm pattern period, corresponding to

a 1.9 resolution gain and **(d)** the spectrum of the whole field of view. Two issues highlight the importance of this figure: The reconstructions using only the Wiener filter are not optimal, the bead with 2.4 resolution gain features a petal-like artifact as consequence of its very patchy power spectrum. For the 1.9 resolution gain no such artifacts occur because the overlap between the central and extended domains is larger and generates a less patchy spectrum, nevertheless, due to the out-of-focus background in the wide-field image, the background in the reconstruction contains features that are not there originally. After applying the apodization function to the spectra of the Wiener filtered reconstructions and empirically adjusting the parameters *a*, *b* and *c,* it is possible to diminish the aforementioned artifacts and improve the reconstructions as demonstrated in the Wiener×Apodization images. Finally, our approach (RL reconstruction) offers a straightforward artifact-minimized reconstruction, which is comparable to the one carried out by the Wiener×Apodization method. The black circle on the spectrums delimits the cut-off frequency of our detection objective. Log_2 was applied on the spectrums for better visualization. Scale bar: 100 nm.

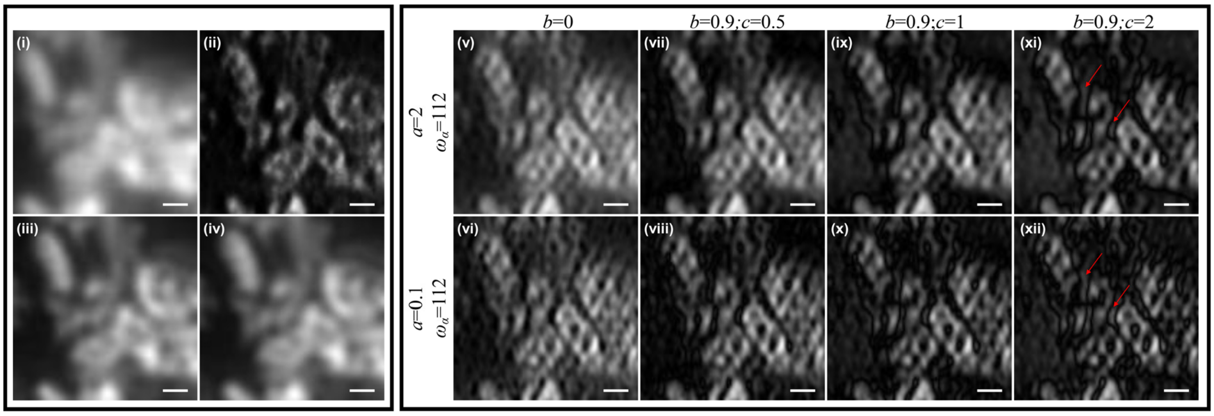

Figure S5 Comparison of Wiener×Apodization and RL reconstructions on images of mitochondria in HepG2 cells. Left side panel: (**i**) Wide-Field, (**ii**) RL reconstruction and deconvolved wide-Field with (**iii**) RL (10 iterations) and (**iv**) Wiener algorithms. Right side panel: (**v-xii**) Several Wiener×Apodization reconstructions are displayed as a function of the parameters a, b and c of the apodization function (Fig. S3). Their tuning is necessary to obtain a good reconstruction. For instance, suppression of the central value of the spectrum with b=0.9 (**vii-xii**) provides a better contrast than the images with no suppression, b=0 (**v-vi**). Also the suppressed area of the spectrum, proportional to the c parameter, influences the reconstruction. The images with c=1 (**ix-x**) have their contrast further improved in comparison to the ones with c=0.5 (**vii-viii**), choosing a slightly larger value, c=2, induces artifacts such as the ones pointed by the red arrows in (**xi**). These artifacts are boosted when trying to make the images sharper by emphasizing the high frequency content by setting a=0.1(**xii**). Choosing a reconstruction becomes a user-dependent matter when using the parameter tuning of the apodization function, this issue is avoided with our RL reconstruction method **(ii)**. Scale bar: 500nm.

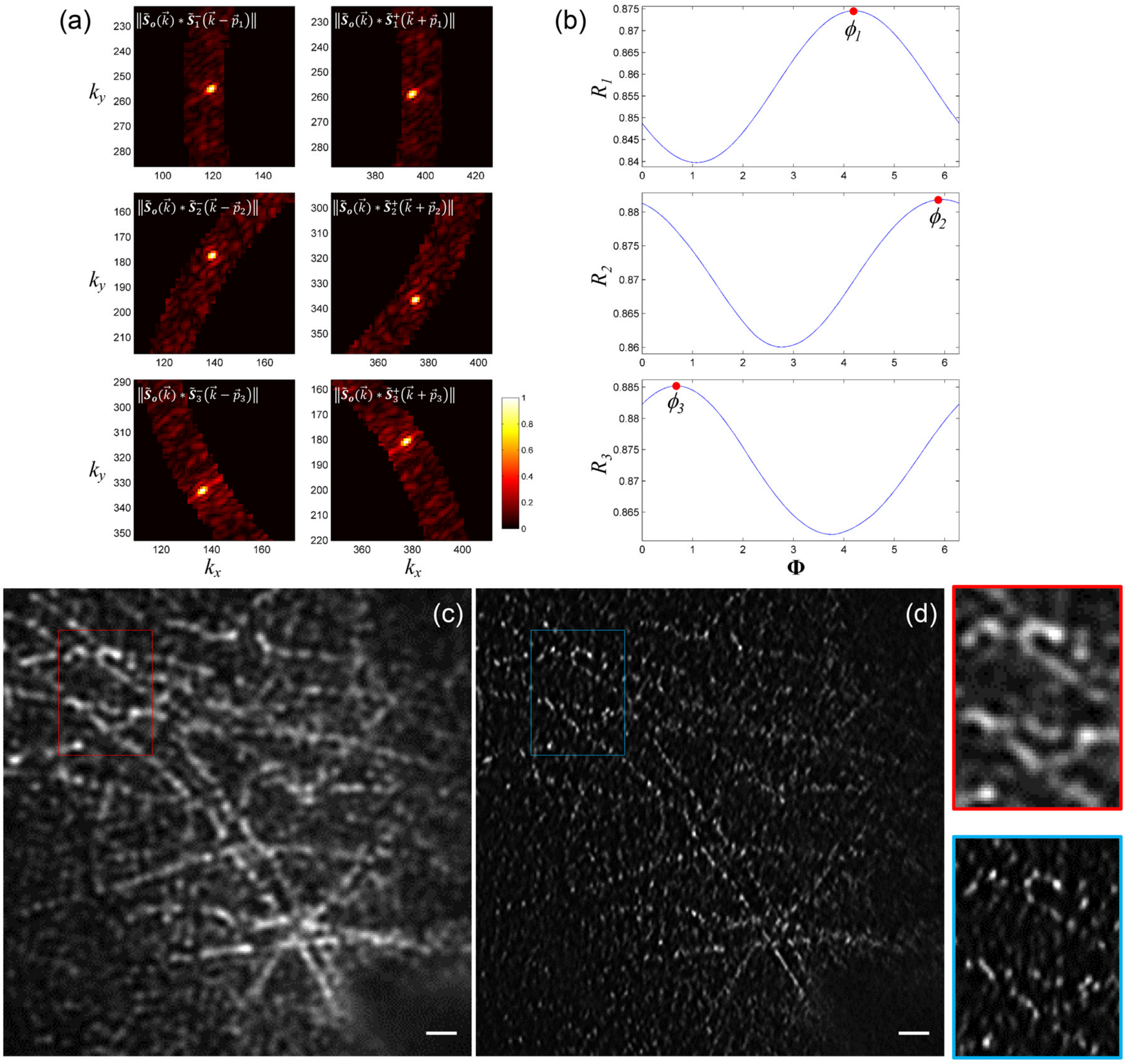

Figure S6 Reconstruction parameters extraction for the HUVE cell data set. (a) Sharp peaks are produced when correlating each of the extended domains with the central one. The position of those peaks determine the spatial frequencies $\pm\vec{p}_j$ of the illumination pattern at each *j* orientation (*j=1,2,3*). **(b)** The initial phases ϕ_j of the illumination pattern are determined from the curves $R_j(\mathbf{\Phi})$. The argument of the maximum in the curves (red dot) defines ϕ_j at each orientation. **(c)** Deconvolved wide-Field with 10 iterations of the Richardson-Lucy algorithm. **(d)** Super-resolved image obtained through our reconstruction method using the initial and continued deconvolution

steps. Magnifications of the selected areas in the red and blue boxes are shown in the rightmost column. Scale bar: 1 μm.

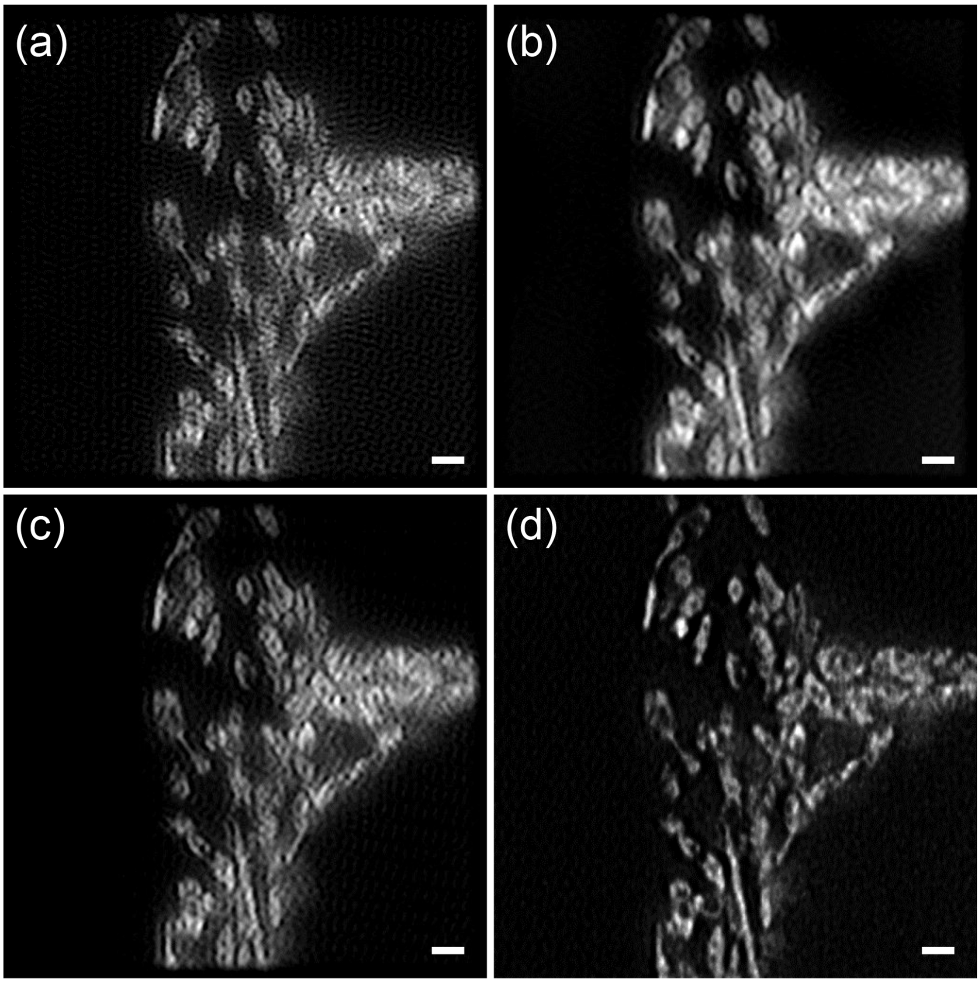

Figure S7 HepG2 data set reconstructed with an open source implementation and with our method. **(a)** , **(b)** and **(c)** images reconstructed with the ImageJ/Fiji plug-in fairSIM [4]. For each of these reconstructions a different set of tunable parameters was used (Table S1). Optimization of the reconstruction was performed by trying out several parameter sets offered by the plug-in to tune the Wiener filter and the apodization function. About 9 different sets were used. To our judgement the best reconstructions the plug-in could produce are the ones in (b) and (c). Notice that in (b) there are less periodic artifacts though less resolution gain than in (c). In (c) the resolution is higher but artifact occurrence is obvious. On the other hand **(d)** the image generated with our RL reconstruction produces, without the need of any parameter tuning, an image with high contrast/resolution and no visible artifacts. Scale bar: 1 μm.

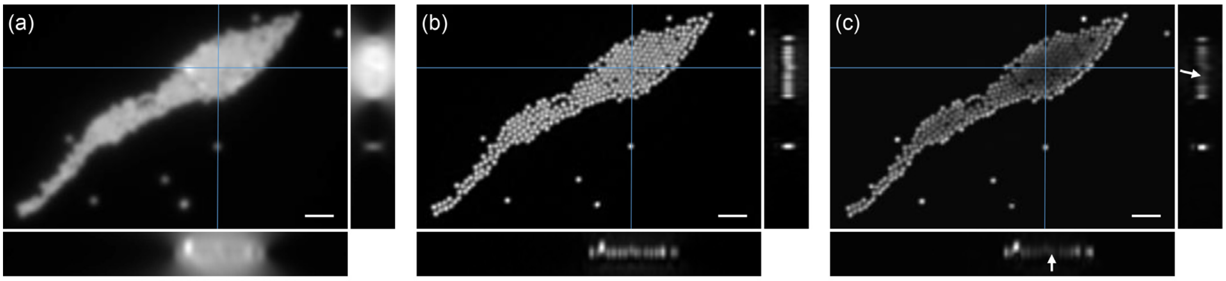

Figure S8 2D vs 3D initial deconvolution for 3D-SIM stacks. Applying a 3D initial deconvolution to data illuminated with a structured pattern produces artifacts that, in order to be avoided, might require the use of 3D spatially variant PSF. This situation is demonstrated with a 3D stack of 100 nm fluorescent beads imaged in an OMX commercial set-up. Orthogonal view of: **(a)** Wide field. **(b)** 3D-SIM reconstruction applying our 2D initial deconvolution approach. **(c)** 3D-SIM reconstruction applying a 3D initial deconvolution. The 3D initial deconvolution was applied to the 5 stacks produced at each phase step, i.e. all the planes comprising a given stack have the same illumination phase. Reconstructions were carried using the 3D-SIM equations in [2]. Beads in (b) are properly reconstructed with no artifacts present in the image. In contrast, the reconstruction in (c) displays an intensity variation across the whole field of view, reaching a dramatic reduction in the center of the beads cluster (white arrows). Such situation points out to the need of a 3D spatially variant PSF to compensate for those variations. To avoid such complication in the initial deconvolution and keep our pipeline robust we stick to a 2D deconvolution, which as observed in (b) does not lead to any deconvolution related artifacts. Scale bar: 1 µm.

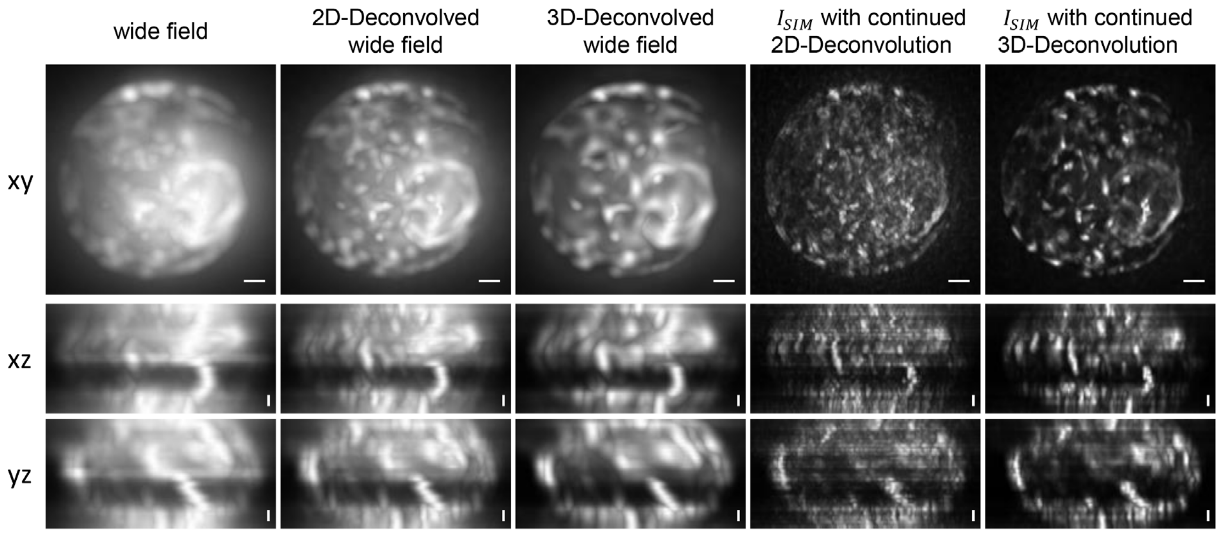

Figure S9 Maximum Projections in xy, xz and yz planes of a Yeast 3D stack. The images display the resolution enhancement when applying a plane-by-plane 2D deconvolution or a 3D deconvolution to the wide field image. Although the 2D deconvolution does not efficiently eliminate the out-of-focus background it emphasizes the in-focus signals over it. The 3D deconvolution allows easier identification of structures along the z-axis. In the same manner, a 3D continued deconvolution can be applied to the plane-by-plane 2D-SIM reconstruction to improve the axial resolution in comparison to the 2D continued deconvolution. Images were acquired with our set-up. Scale bar: 500 nm.

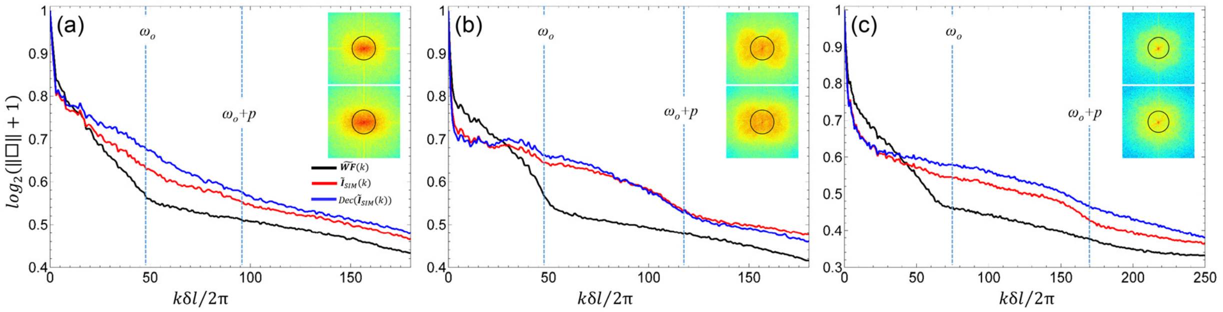

Figure S10 The continued deconvolution enhances contrast by re-distributing the spectra of the reconstruction. The circular average of the power spectrum of the wide field (black line), RL reconstruction (I_{SIM}, red line) and I_{SIM} with continued deconvolution (blue line) is presented for three different image data sets: **(a)** HepG2 cell, **(b)** HUVE cell and **(c)** TIRF-SIM microtubules. The complete power spectrum of the reconstruction is shown in the top left corner insets. Upper box: I_{SIM}. Lower box: deconvolved I_{SIM}. Vertical lines indicate the cut-off frequency of the wide field (ω_o) and the cut-off frequency (ω_o+p) of the SIM reconstructions, with p denoting the norm of the pattern spatial frequency. Constants δ and l in the x-axis label denote the pixel-size and the image length in pixels. Plots (a) and (b) show that the blue curve lies above the red curve in the range of middle and high spatial frequencies, only to converge to the same noise baseline passing the frequency ω_o+p. This situation indicates a contrast improvement of the deconvolved I_{SIM} image rather than an increase in frequencies beyond ω_o+p. In plot (c) the blue curve is found above the red one beyond ω_o+p. Such case might indicate a slight resolution gain due to the non-negativity constraint of the RL algorithm [5,6].

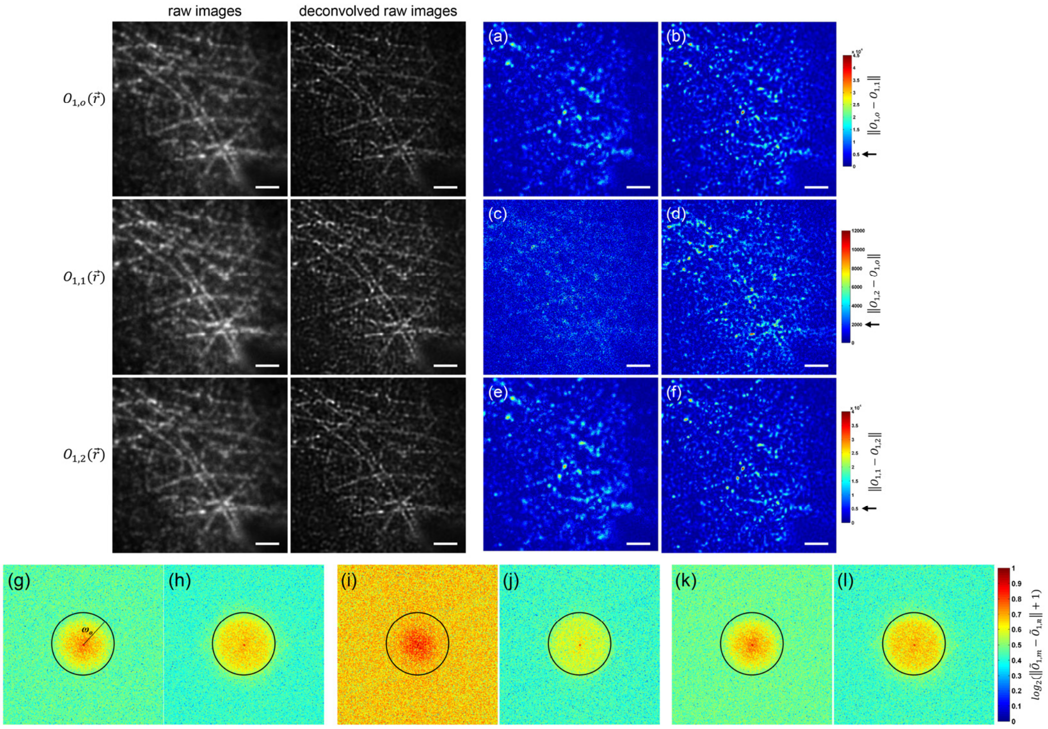

Figure S11 The initial deconvolution enhances the extraction of high frequencies. The extended domains $\tilde{\boldsymbol{S}}_j^{\pm}$ that carry the high spatial frequencies are formed by the differences between the $\widetilde{\boldsymbol{O}}_{j,m}$ images acquired with the different m phases of the pattern (Eq. 7). The initial deconvolution increases the modulation, i.e. it increases these differences over a given background. These represents a two-fold advantage, because it enhances the extraction of high frequencies and also allows the recovery of features in the image where the modulation in the raw images was not enough to represent a resolution gain. To illustrate this, the absolute value of the intensity differences $O_{1,m} - O_{1,n}$ has been calculated for the raw images in **(a)** , **(c)**, and **(e)** and also for the deconvolved raw images in **(b)** , **(d)**, and **(f)**. Respectively their power spectrum is shown in **(g)**, **(i)** , **(k)** and **(h)**, **(j)** , **(l)** . Consistently one can observe that the background level (black arrows) remains the same for each pair (a)-(b), (c)-(d) and (e)-(f), but using the deconvolved raw images to calculate the differences allows to salvage signals from background levels in comparison to the differences calculated only with the raw images. This effect is very obvious in (c)-(d), since in (c)

image features are barely recognizable, whereas in (d) many features have been successfully recovered and lie above background levels. Such recovered features represent more information that can be incorporated into the extended domains $\tilde{\mathbf{S}}_j^{\pm}$ as seen in the spectra (i)-(j). Hence the initial deconvolution allows the recovery of image features that might be buried in the background due to a low pattern modulation, this is specially useful when using resolution gains ≥ 2, since this involves illumination patterns with a spatial frequency in the limits of the cut-off frequency ω_o, situation that inherently leads to low modulations. Scale bar: 2 μm.

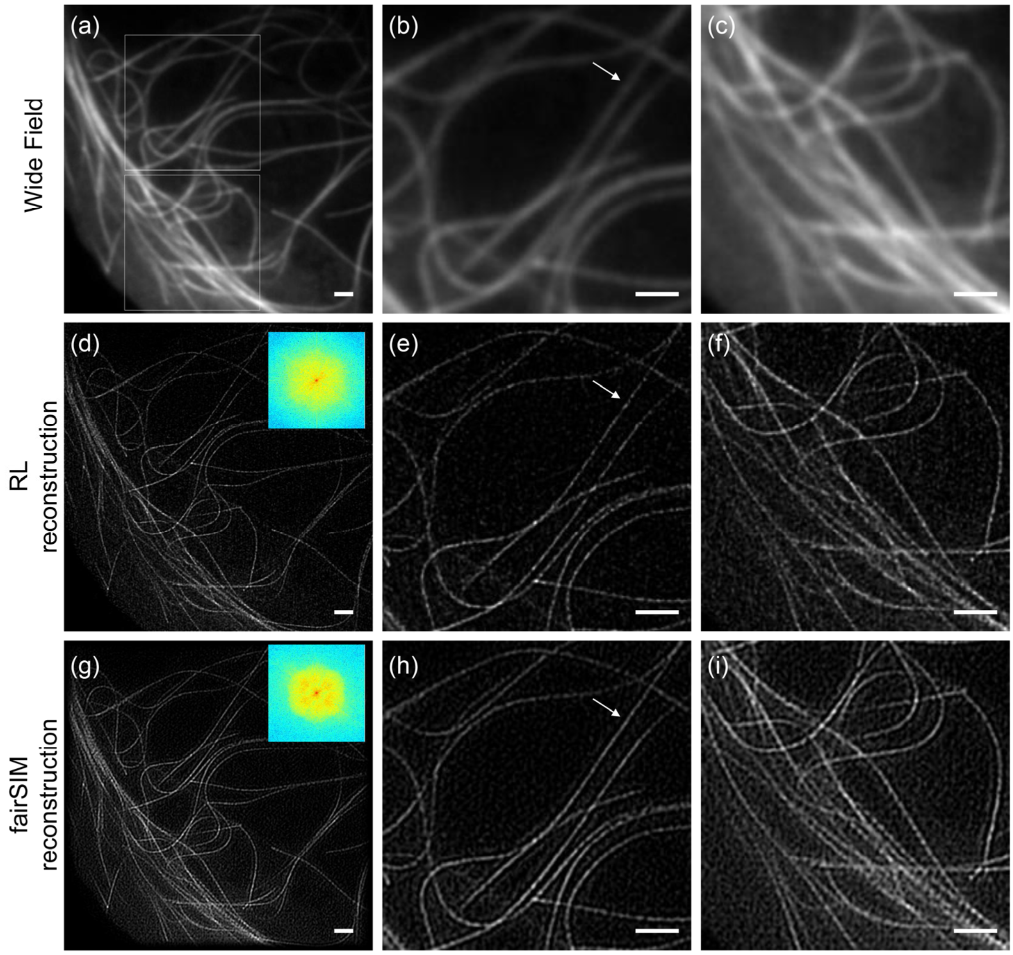

Figure S12 Testing RL reconstruction with an external data set. A TIRF-SIM data set of tubulin emitting at 525 nm was reconstructed with the fairSIM implementation and our two step deconvolution approach. The first row shows the **(a)** wide field and expansions of the its **(b)** upper and **(c)** lower halves. Similarly the second and third row display the **(d)** RL and **(g)** fairSIM reconstructions, with their corresponding power spectra on the top left corner inset. The region in (b) has a cleaner background in contrast with (c) which presents a high background signal. These two regions are used to compare slight differences between the two reconstruction approaches for this particular image set. Firstly, microtubules in region (h) appear more continuous and with

higher intensity than in (e), to the point that for some microtubules (white arrows) these two features do not match with the intensities displayed in the wide field (b). This difference can be explained if one observes that the spectra of (d) seems more evened out in comparison to the spectra of (g) which has more emphasis on the low central frequencies and a ring-like area of dimmed middle range frequencies. A second difference is noticed in the high background areas (c), e.g. due to the initial deconvolution the reconstruction in (f) appears to have a cleaner background than (i). In spite of these differences, both reconstructions are practically the same in terms of overall structures and features present in the images. The data set as well as the input parameters for the fairSIM reconstruction where downloaded from the fairSIM website [4]. These parameters along with the parameters extracted by our reconstruction approach can be found in Table S2. Scale bar: 1 µm.

Reconstruction	$Period_1$ (nm) / angle (°)	$Period_2$ (nm) / angle(°)	$Period_3$ (nm) / angle(°)	strength	FWHM	Wiener Parameter	Apodization cut-off
(a)fairSIM*	305.57 / 0.04	293.94 / -26.99	292.28 / 26.74	0.99	0.15	0.05	2
(b)fairSIM*	305.57 / 0.04	293.94 / -26.99	292.28 / 26.74	0.99	1.2	0.2	1.5
(c) fairSIM*	305.57 / 0.04	293.94 / -26.99	292.28 / 26.74	0.99	0.15	0.2	2
(d) RL Reconstruction*†	307.20 / 0	295.71 / -26.82	293.086 / 26.5651	_	_	_	_

*PSF of NA 1 and emission wavelength of 515 nm.
† 10 iterations for the initial deconvolution. 5 iterations for the continued deconvolution.

Table S1 Reconstruction parameters of the HepG2 data set (Fig. S7). The step size of the pattern phases in all reconstructions is 120°. The initial phase is not presented since it is a relative value whose value depends on the chosen range of angles.

Reconstruction	$Period_1$ (nm) / angle (°)	$Period_2$ (nm) / angle(°)	$Period_2$ (nm) / angle(°)	strength	FWHM	Wiener Parameter	Apodization cut-off
fairSIM*	179.70 / -89.26	178.61 / -28.90	178.26 / 30.50	_	_	0.1	2
RL Reconstruction*†	180.18 / -89.04	178.83 / -28.84	178.10 / 30.53	_	_	_	_

*PSF of NA 1.49 and emission wavelength of 525 nm.
† 10 iterations for the initial deconvolution. 5 iterations for the continued deconvolution.

Table S2 Reconstruction parameters of the TIRF-SIM data set (Fig. S12). The step size of the pattern phases in all reconstructions is 120°. The initial phase is not presented since it is a relative value whose value depends on the chosen range of angles. As suggested by reference [4] a correction of 405 was applied to the raw images to compensate for camera noise.

1. Greger, K., Swoger, J. & Stelzer, E. H. K. Basic building units and properties of a fluorescence single plane illumination microscope. *Rev. Sci. Instrum.* **78,** 23705 (2007).

2. Gustafsson, M. G. L. *et al.* Three-dimensional resolution doubling in wide-field fluorescence microscopy by structured illumination. *Biophys. J.* **94,** 4957–70 (2008).

3. Wicker, K., Mandula, O., Best, G., Fiolka, R. & Heintzmann, R. Phase optimisation for structured illumination microscopy. *Opt. Express* **21,** 2032–2049 (2013).

4. Müller, M., Mönkemöller, V., Hennig, S., Hübner, W. & Huser, T. Open-source image reconstruction of super-resolution structured illumination microscopy data in ImageJ. *Nat. Commun.* **7,** 10980 (2016).

5. Sementilli, P. J., Hunt, B. R. & Nadar, M. S. Analysis of the limit to superresolution in incoherent imaging. *J. Opt. Soc. Am. A* **10,** 2265 (1993).

6. VERVEER, P. J., GEMKOW, M. J. & JOVIN, T. M. A comparison of image restoration approaches applied to three-dimensional confocal and wide-field fluorescence microscopy. *J. Microsc.* **193,** 50–61 (1999).
